# Ueber Die Zweckmässigste Einrichtung der Feld Hospitäler

**Published:** 1801-12

**Authors:** 


### CRITICAL RETROSPECT

### OF

### MEDICAL AND PHYSICAL LITERATURE.

---

*Ueber Die Zweckmässigste Einrichtung der Feld Hospitäler;* i. e. On the moſt proper Arrangement of Field Hoſpitals, by Dr. G. P. MICHÆLIS, late Field Phyſician in the Electoral Brunſwick Lunebourg Services; with an engraving. Gottingen, 1801, Dieterich, pp. 520, in octavo.

THE author of the preſent work has a juſt claim to the approbation of the community, for having undertaken the difficult taſk of thoroughly examining the condition of field hoſpitals, of pointing out the defects to which they are ſtill expoſed, and of making ſuch propoſals, with reſpect to their improvement, as are fit for being properly executed; in which attempt he has perfectly ſucceeded, ſhowing, at the ſame time, a moſt accurate knowledge of the ſtate of military hoſpitals; and we have no doubt but this work will prove of great ſervice to military phyſicians. Beſides an introduction, the materials of the work are contained in *three* ſections. In the introduction, the author juſtly reprehends the neglect which inſtitutions of that kind generally experience in time of peace, where where no meafures are taken, no preparations made, by which, at the commencement of a war, hofpitals might be immediately arranged, and phyficians provided; indeed, it frequently happens, that the plan for the maintenance of the fick and wounded is not even fettled when the army keeps the field, which circumftance, however difadvantageous to the intereft of the ftate, is witneffed in the hiftory of the prefent war. He farther difcuffes, whether it contributes to the benefit of the hofpital to unite medicine and furgery in one perfon, or whether it is better to have thefe two branches of the healing art exercifed by feparate perfons? the laft of which the author is inclined to prefer. But upon nearer confideration it will be found lefs practicable, becaufe the hofpitals being fometimes only filled with fick people, at other times with wounded, an equal number of phyficians and of furgeons would be required; a circumftance which, from the great additional expence of maintaining a double body of men, would prove extremely burdenfome to the ftate, not being of proportional utility, particularly as we do not fee, why a perfon fhould not by proper application become equally fkilled in medicine as well as in furgery. The author alfo fpeaks of the rank that ought to be affigned to army furgeons, and of a proper drefs, to make them more refpected than they generally are.

First Section. *Of the Arrangement of Hospitals.* Here he fhows the advantage of regimental hofpitals, in which the patients are in general better provided for than in field hofpitals, where the number of patients frequently increafe to fuch a degree, as to make it impoffible to pay proper attention to each individual. The fituation of an army in the field will however rarely allow the eftablifhment of feparate hofpitals, neither regimental nor thofe which, according to the propofal of the author, fhould be eftablifhed, about a mile diftant from the camp, for cafes of emergency; becaufe, when the army is in fight of the enemy, and expofed to tumultary movements, retreats, &c. an hofpital at fuch a place is frequently difturbed, and fubject to unavoidable mifchief and different vexations; moreover, it is known that in thofe cafes the number of waggons is hardly fufficient to convey the wounded, how then fhould the fick in the hofpitals be brought away? The advantages, however, that refult from regimental hofpitals, &c. ought not to be difregarded in winter quarters, or at the time of an armiftice. For the better conveyance of the fick, the author propofes that each regiment fhould be furnifhed with two waggons, each being large enough for containing eight men.—*On common Hospitals,* which he divides into *three* claffes, viz. ambulatory hofpitals, depot hofpitals, and chief hofpitals.—*On the Situation and Use of the Chief Hospital.*—It fhould be fituated at a proper diftance from the army; and each feparate army, detached from the great army, fhould have a chief hofpital of its own, capable of following it in the different movements; each fortified place fhould likewife be provided with a chief hofpital. The fecurity of the places for the eftablifhment of chief hofpitals, is beft known and afcertained afcertained by the chief general. Confiderable towns or cities are beft calculated for the eftablifhment of chief hofpitals; but lefs proper for hofpitals are fortreffes expofed to a fiege. The chief hofpital fhould not be above fix German miles diftant from the ambulatory hofpitals; the patients not too much crouded in any hofpital, and, if poffible, placed near a river or canal, whereby the conveyance of the patients becomes more convenient and lefs expenfive. Lingering patients, &c. ought to be fent back to the garrifon hofpitals. Mr. Michælis rejects the feparation of the patients according to their refpective forms of difeafes; and he expreffes the humane wifh, that the refuge of the fick and wounded might be declared neutral; but to fuch a degree of humanity in wars we are not yet arrived.—*On the Use and Situation of ambulatory Hospitals.* They are deftined for the reception of the fick and wounded from the regiments; but no patients ought to be kept there too long, excepting thofe who are dangeroufly ill, in order to have fufficient room for the new arrivals; their fituation ought to be as near behind the army as poffible, and for better communication with the chief hofpital, near a river or canal, &c.—*On the Depot Hospitals.—On the Hospitals in besieged Towns.—On the Choice of the Building for an Hospital.—On the inner Disposition of an Hospital.—On the Wards.—On the Means of renewing and correcting the Air in the Wards.* A great many propofals have been made for that purpofe, but for the moft part too compound and artificial to be properly executed in field hofpitals, where the moft poffible fimplicity fhould be obferved, on account of their being expofed to continual movement. Cleanlinefs is the firft requifite for preventing the air being corrupted, and if that be properly attended to, the opening of the doors and windows in the morning, noon, and before fun-fet, is undoubtedly the moft fimple and beft ventilator. It is always defirable to place very weak patients in the upper ftory, pure air having there a freer accefs. Mr. Michælis rejects upon good grounds, fumigations with refinous and fcented fubftances for the purpofe of purifying the air, as it is more corrupted than corrected by them. The burning of gun-powder, though recommended by the author, feems to be exceptionable, as it only imparts a falutary motion to the air. The evaporation of muriatic acid, fo much praifed by fome, does not anfwer the purpofe, becaufe it frequently occafioned a vehement coughing to the patients. For renewing the air in winter time the author propofes ftoves with Mr. Salmon's tubes; but it may be fuggefted, that all fuch propofals can only be executed in field hofpitals, more fettled at a place than they are frequently allowed.—*On the Position of the Bedsteads.* They ought not to be too clofely put together.—*On the Apartments for the Reception and Cleansing of the Patients.* At the entrance of the hofpitals one or two fpacious apartments fhould be appointed, where the patients are to be received and cleaned, where they ought to change their uniforms with the dreffes of the hofpital, and whence they are to be diftributed into the different wards by the attending phyficians.—*On the Apartment for the Subaltern Officer*

*upon Duty.—On the proper Place for Privies.—On the Tea Kitchen.—On the Dead's Chamber.—On the Apartments for the Operations, Bandages, and Surgeons.—On the Place for the Apothecary's Shop.—On the Kitchen.—On the Buildings for the Magazine of the Hospital.—On the Lodgings of the Persons attending the Hospital.* All the perſons belonging to the hoſpital ought to live in it, and not have lodgings at ſome diſtance from the hoſpital.—*On the Buildings for the recovering Persons.* Proper buildings at ſome diſtance from the hoſpital ought to be appointed for the perſons recovering, and they ought to be kept under military and medical inſpection.—*On the Utensils belonging to the Hospitals.—On the Bedsteads and similar Furniture.*—The author deſcribes here the ill conſequences of bedding the patients on the ground; the bedſteads, however, ought to be as ſimple as poſſible, for preventing them becoming an incumbrance to the army.—*On the Straw Sacks.*—They ought to be kept clean, and the hoſpital provided with a ſufficient quantity of them; and for 1000 patients, 2000 or 2500 ſtraw ſacks ought to be provided. The author rejects the uſe of quilts ſtuffed with horſe or cow-hair, as being reſervoirs for every kind of miaſmata, and particularly as contributing to the propagation of the itch.—*On Bed-cloths and Coverlets.—On Bed Skreens.* Only prope for garriſon hoſpitals.—*On the Dress of the Patients.* The patients ought to be provided immediately after entrance into the hoſpital with a dreſs belonging to the hoſpital, for which he propoſes trouſers and ſhort coats, made of ſtrong linen or fuſtian. A ſufficient ſtock of ſhirts and ſtockings ſhould likewiſe be provided for in field hoſpitals.—*On the Vessels for Eating ana Drinking.* A plate, a baſon, and a trikel, with a ſpoon of tin or white iron.—*On the Utensils for Medical Use.—On other Furniture necessary to the Patient.—On Close-stools.—On the urinary Vessels, and different other Furniture belonging to an Hospital.—On the Train and Carriages belonging to an Hospital.—On the Carriages for transporting the patients.* It is to be regretted that except the Engliſh and Ruſſian, no other armies are provided with carriages properly calculated for the conveyance of the ſick and wounded, however great the advantages are which hence reſult for the welfare of the patients. It is indeed ſhocking for a feeling heart to ſee the poor ſick and wounded warriors dragged along on open waggons, that are taken from the peaſants on the way, in which they are expoſed for days to every ſort of weather. The author highly recommends the carriages introduced in the Engliſh army for the conveyance of the ſick, of which a deſcription and plan are added.

SECOND SECTION. *On the Maintenance of the Patients.* The nouriſhment or food of the patients deſerves particular attention, as it is frequently ſufficient by itſelf to reſtore health, if it is properly choſen and well prepared.—*On the Victuals for the Patients.* The author reprehends here the cuſtom of always offering animal food to patients, particularly to thoſe who labour under a putrid ſtate; he particularly recommends the diet obſerved in the Auſtrian and French hoſpitals.—*On Bread and Meat.* He prefers beef, and and for weaker patients veal, above the reft.—*On Vegetable Food.*—*On the Breakfast.* For which he recommends warm beer with fome white bread, and for weaker patients with the yolk of an egg.—*On Supper.* It ought to confift of gruel of flour and bread.—*On the Drink.* The author greatly rejects the decoctions and infufions introduced into many hofpitals, and promifcuoufly given to every patient; he prefers pure water; which he recomends to be cooled with fnow and ice in putrid and nervous fevers, a practice that is in moft cafes very exceptionable. The mixture of vinegar and water, which the author is inclined to recommend as the common beverage in hofpitals, is liable to many objections.—*On Tobacco.*—*On the Rations. On the Advantages in purchasing the Necessaries and Provisions for an Hospital.*—*On the washing of the Linen of the Patients.*—*On the Transport of the Patients into the Hospital, and their Reception.*—*On the Means of making the Transport less difficult.*—*On the Provisions during the Transport.*—*On the Convalescents.*—*On the Invalids.*—*On the Burial of the Dead.* Mr. M. propofes to take the dead, with the ftraw facks on which they died, immediately out of the wards, but to place them in a proper apartment that is heated in winter, in which they ought to be obferved from time to time, and not to bury them before they have been laying there about twenty-four hours, and infpected by a phyfician.

Third Section. *On the Persons belonging to the Hospital, and on the Direction.* The affairs of the hofpitals fhould not be conducted by a fingle man, on account of their being of fo different a nature, that it is impoffible one man fhould be able to make proper regulations, which require an accurate knowledge, œconomical as well as medical. The direction of the hofpitals fhould therefore be intrufted to a committee.—*On the Choice of the Persons belonging to the Direction.*—*On the first Officer and his Qualities.*—*On the first Physician and Surgeon, and their respective Qualities.*—*On the Choice of the first Steward and his Qualities.*—*On the Choice of the rest of Persons in the Service of the Hospital.*—*On the Duties of the Persons belonging to the Direction of the Hospital.*—*On the Duties of the rest.*—*On the Means of obtaining good Subaltern Surgeons, and on their Medical Education.* A paragraph containing feveral propofals worthy the attention of the public. Such are the contents of a book, which every where difplays the profoundeft knowledge and experience of the author, in the actual ftate of military hofpitals, and which will afford the greateft fatisfaction to all who confult it.

